# *Krüppel homolog 1* mediates juvenile hormone action to suppress photoperiodic reproductive diapause-related phenotypes in the female *Chrysoperla nipponensis* (Neuroptera: Chrysopidae)

**DOI:** 10.1093/jisesa/ieaf027

**Published:** 2025-03-21

**Authors:** Haiyi Huang, Dandan Li, Minghui Xu, Shaofeng Zhong, Shaoye Liu, Xingke Gao, Yongyu Xu, Zhenzhen Chen

**Affiliations:** College of Plant Protection, State Key Laboratory of Wheat Improvement, Shandong Agricultural University, Tai’an, China; College of Plant Protection, State Key Laboratory of Wheat Improvement, Shandong Agricultural University, Tai’an, China; College of Plant Protection, State Key Laboratory of Wheat Improvement, Shandong Agricultural University, Tai’an, China; College of Plant Protection, State Key Laboratory of Wheat Improvement, Shandong Agricultural University, Tai’an, China; College of Plant Protection, State Key Laboratory of Wheat Improvement, Shandong Agricultural University, Tai’an, China; College of Plant Protection, State Key Laboratory of Wheat Improvement, Shandong Agricultural University, Tai’an, China; College of Plant Protection, State Key Laboratory of Wheat Improvement, Shandong Agricultural University, Tai’an, China; College of Plant Protection, State Key Laboratory of Wheat Improvement, Shandong Agricultural University, Tai’an, China

**Keywords:** green lacewing, juvenile hormone, reproductive diapause, *Krüppel-homolog 1*, photoperiod

## Abstract

Juvenile hormone (JH) has been revealed to be a critical factor in regulating photoperiod reproductive diapause in various insect species, however, little information is known about the detailed mechanisms. In this study, we investigated the roles of JH signaling in photoperiod reproductive diapause in a green lacewing, *Chrysoperla nipponensis* (Okamoto), which is a potentially important biological control predator. Our results showed that the short-day condition induces a diapause state including JH synthesis suppression, ovarian development arrest, and triglyceride accumulation. The interference of JH response genes, *Krüppel homolog 1* (*Kr-h1*), in reproductive females exhibited a diapause-related phenotype such as ovarian development arrest and larger triglyceride storage. Exogenous JH III suppresses diapause to promote ovarian development and inhibit triglyceride synthesis. However, exogenous JH III fails to rescue the *Kr-h1*-silenced phenotype. Accordingly, our results demonstrate the critical role of *Kr-h1* in regulating JH signaling to promote reproduction.

## Introduction

Short photoperiod signals a harbinger of winter, and insects usually use day-length to program their diapause for overwintering (Denlinger 2002). Diapause is a programmed, stage-specific developmental arrest or delay that is often used to avoid unfavorable seasons (Denlinger 2022). Reproductive diapause in female adults is usually characterized by stagnation of vitellogenesis and oocyte development, phenotypically as a completely undeveloped ovary with no vitelline deposition, and finally by suppression of egg-laying behavior (Denlinger et al. 2012). Besides, studies on *Culex pipiens* have shown that diapause was accompanied by the sequestration of fat reserves in female, in addition to ovarian development arrest (Sim and Denlinger 2008). Juvenile hormone (JH) is the primary hormone that control insect reproduction, and most of the early work on adult diapause hormonal control mainly focused on JH.

Earlier works on the relationship between JH and diapause were initiated by applying JHs or juvenile hormone analogues (JHA) to terminate *Leptinotarsa decemlineata* adult diapause (de Kort 1990, Denlinger et al. 2012). Nevertheless, current studies on the reproductive diapause mainly focused more on the role of JH synthesis (Gao et al. 2022a) and JH response genes (Guo et al. 2021). *Krüppel homolog 1* (*Kr-h1*), one of the early JH response genes (Minakuchi et al. 2009), acts downstream of JH signaling (Minakuchi et al. 2008) and plays a vital role in reproductive diapause (Guo et al. 2021). Typically, silencing of *Kr-h1* leads to the blockage of vitellogenesis and the cessation of oocyte development during reproduction, such as studies on *Locusta migratoria* (Song et al. 2014) and *Periplaneta americana* (Zhu et al. 2020). In addition, a previous study had shown that *Kr-h1* controls lipid accumulation and triglyceride content in summer diapause beetles, *Colaphellus bowringi* (Guo et al. 2021).

The green lacewing, *Chrysoperla nipponensis* (Neuroptera: Chrysopidae), is one of the predominant predatory enemies in the field throughout China. *C. nipponensis* larva is commonly known as an aphid lion to attack numerous agricultural and forest pests, including aphids, mites, and both eggs and young larvae of many Lepidoptera pests (Xu et al. 1999). Similar to other *Chrysoperla* predator species that undergo facultative reproductive diapause under short-day conditions (Chang et al. 1995), *C. nipponensis* overwinters as an adult stage (Xu et al. 2002, Chen et al. 2017). *C. nipponensis* is a good model for studying the regulation mechanism of adult reproductive diapause, since females were reproductive under long-day condition and short-day condition lead to diapause, and the diapause phenotype is easy to observe, ie, diapause individuals with yellow body color, and nondiapause individuals with green body color (Chen et al. 2017, 2023, Liu et al. 2024). Previous study demonstrated significant accumulation of total lipid and triglyceride content during diapause in intact *C. nipponensis* females, and combined transcriptomic and proteomic analyses have shown that JH pathway and *Kr-h1* may be essential in the maintenance of adult reproductive diapause (Chen et al. 2023). In addition, manipulating the diapausing stage of *C. nipponensis* can be used for storage or long-distance transport (Denlinger 2008). However, the regulatory mechanisms underlying reproductive diapause of *C. nipponensis* remain unexplored.

The purpose of this study was to explore the role of JH and *Kr-h1* in the reproductive diapause of *C. nipponensis* females. In the present study, we generated diapaused female *C. nipponensis* and characterized its phenotypic effects, and then analyzed the differentially expressed JH-related genes between reproduction and diapause. Finally, RNA interference (RNAi) and exogenous JH rescue was used to determine the functions of *Kr-h1* on reproductive females. Our work highlights that *Kr-h1* mediates JH action to suppress photoperiod reproductive diapause in *C. nipponensis* females. This study will be beneficial for the potential applications in mass artificial storage of diapausing predatory insects, such as *C. nipponensis.*

## Materials and Methods

### Insect Rearing

*Chrysoperla nipponensis* was collected from a plant nursery in Taian, Shandong Province, China (36°15′N, 116°59′E), and maintained in an environmental chamber (RSZ intelligent artificial climate chamber, Changzhou Guohua, Jiangsu province) with 25 ± 1 °C temperature, 70 ± 5% relative humidity (RH), and long photoperiod of 15:9 h (L:D). Both eggs and larvae were kept in glass tubes (1 × 7 cm) and reared on *Megoura japonica*. Adults were paired in glass cylinders (18 × 9 cm) and supplied with a dry powdered mixture of yeast–sugar (yeast:sugar = 10:8) and a 10% honey–water solution as food. After two generations, eggs laid within a 24 h were collected and were randomly divided into two groups. The diapause population was kept at 25 ± 1°C, 70 ± 5% RH, and short photoperiod of 9:15 h (L:D), whereas the reproduction population was kept under the conditions of long photoperiod of 15:9 h (L:D).

### JH Titer Measurement

The female lacewings, reared under long-day photoperiod (LD) and short-day photoperiod (SD) conditions, were collected for JH titer measurement at 1, 3, 5, and 10 d after emergence. Seven individuals as one replicate, 3 biological replicates per treatment. The lacewings were washed with ddH_2_O, and then placed in 1.5 ml tubes that containing 0.1 ml acetonitrile and 0.1 ml 0.9% (w/v) sodium chloride solution. The samples were ground by the electric pestle for 1 min after frozen in liquid nitrogen. The homogenate was extracted twice with 1 ml hexane following vigorous vortex and centrifuged at 2,500 × *g* for 5 min. The supernatant was transferred to a new 1.5 ml tube and dried under a stream of N_2_. The dry extract was then reconstituted with 25 µl of a solution and injected into the GC-MS/MS system. The GC parameters and triple-quadrupole MS parameters were set according to a rapid quantitative assay method, and the specific selected-reaction monitoring transition of JH III was 85.1 (59.1) under 10 collision energy (Kai et al. 2018). The GC-MS/MS system consists of Agilent 7890B GC (Agilent, USA) and Agilent 7000D MS (Agilent, USA). Gas chromatographic column was HP-5 MS 5% diphenyl–95% dimethyl polysiloxane capillary column (30.0 m × 250 μm × 0.25 μm, Agilent Technologies). The ion transfer capillary temperature was maintained at 280 °C. The mass spectrometer operates in EI mode (70 eV) with an injection current of 50 A.

### Triglyceride Content Measurements

The adult was washed with ddH_2_O and placed in a 1.5 ml tube containing absolute ethyl alcohol (insect weight (g):alcohol (ml) = 1:9). Eight to 12 individuals as 1 replicate, 3 replicates per treatment. Then the samples were centrifuged at 6,000 rpm (3,743 × *g*) for 10 min after homogenization. Triglyceride content (in upper layer) was determined using a Triglycerides Assay Kit (Nanjing Jiancheng Institute, Nanjing, China) according to the manufacturer’s instructions. The absorbance of the solution at 510 nm was measured by Molecular Devices SpectraMax I3× (PerkinElmer, USA).

### Total RNA Extraction, cDNA Synthesis, and Real-Time qRT-PCR Analysis

Total RNA was extracted from fat body or intact lacewing using MiniBEST Universal RNA Extraction Kit (Takara, Japan) following the manufacturer’s instructions. RNA integrity was estimated by 1% agarose gel electrophoresis. The concentration and purity of RNA were examined by NanoDrop One spectrophotometer (Thermo Scientific, USA). The HiScript II Q RT SuperMix for qPCR (+gDNA wipers; Vazyme, Nanjing, China) was used for the first-strand cDNA synthesis following the manufacturer’s instructions. ChamQ SYBR qPCR Master Mix (Vazyme, Nanjing, China) and the Roche LightCycler 96 instrument (Bio-Rad, USA) were used for qPCR reaction. A standard curve was prepared based on a gradient-based cDNA dilution, and specific primers with appropriate amplification efficiency (0.95 to 1.05) were determined (Table 1). Expression of key genes of JH pathway, JH acid O-methyltransferase (*Jhamt*), *Kr-h1*, and *Vitellogenin* (*Vg*), at 1, 3, 5, and 10 d of intact lacewing after emergence were measured. Expression of *Krh1* and *Vg* on the fat body at 5 and 10 d of lacewing after emergence were measured. The reference gene, *Tubulin (Tub)*, for qPCR analysis was referenced to the previous study (Wang et al. 2020). Five individuals as 1 replicate, 3 replicates per treatment. The relative expression levels of target genes were calculated using the 2^−△△*ct*^ method.

**Table 1.** PCR primers used in this study

Primers	Primer sequences (5′–3′)
*Jhamt*-qF	GGTTATCAAACGGTGGTG
*Jhamt*-qR	TCCGTGTGTATTTGGCA
*Kr-h1*-qF	CTGGTGATACACGTCGTGTTGT
*Kr-h1*-qR	TGCACTGATAGGGATCATCTCG
*Vg*-qF	TACAACAGTCCATTCGCTGCTG
*Vg*-qR	GCATAGGTTGATAGTCGTCAGC
*Tub*-qF	CGGAAACCAGATTGGAGCTAAG
*Tub*-qR	CCAAATGGACCAGAACGTACTG
ds*Kr-h1*-F	TCGACACATGCGTATACATACA
ds*Kr-h1*-R	CAGGTGATGCTTGTTGAGGAT
ds*Kr-h1*-TF	taatacgactcactataggTCGACACATGCGTATACATACA
ds*Kr-h1*-TR	taatacgactcactataggCAGGTGATGCTTGTTGAGGAT
ds*Gfp*-F	GCGACGTAAACGGCCACAAGT
ds*Gfp*-R	GTACAGCTCGTCCATGCCGAG
ds*Gfp*-TF	taatacgactcactataggGCGACGTAAACGGCCACAAGT
ds*Gfp*-TR	taatacgactcactataggGTACAGCTCGTCCATGCCGAG

### RNAi

Double-stranded RNA (dsRNA) of *Kr-h1* was prepared using the corresponding primers (Table 1). The dsRNA was synthesized using a T7 RiboMAX Express RNAi kit (Promega, USA) following the manufacturer’s instructions. The dsRNA quality was determined at 1% agarose gel electrophoresis, and the concentration was measured in a NanoDrop One spectrophotometer (Thermo Scientific, USA). Then, 1 μg of dsRNA was microinjected into the abdomen of newly emerged adult females, and the ovary and fat body were collected 10 d later for phenotypes analysis.

### Exogenous JH Application

The original racemic JH III solution [Purity (HPLC): ≥65%, Sigma-Aldrich, St Louis, MO, USA] was diluted to 10 mg/ml using acetone. To test the function of JH in the regulation of reproductive diapause in *C. nipponensi*s*, 2* µl of JH III dilution was topically applied onto the abdomen of newly emergence adults under SD condition using a hand micro-applicator (Burkard, the United Kingdom). Besides, JH III was also dropped onto the abdomen of *C. nipponensis* with ds*Kr-h1* injected for 3 d. Meanwhile, 2 µl of acetone was dropped as the solvent control.

### Ovarian Size Measurement

Ovaries were carefully removed from the female lacewings. Eight to 12 individuals as 1 replicate, 3 replicates per treatment. The images were collected using a stereo microscope equipped with a Nikon SMZ800 (Nikon, Japan). The images of the ovaries were captured with a Nikon D5100 digital camera (Nikon, Japan), and the apical ovarioles length of the ovary was measured with Adobe Photoshop 13.0.1 (Supplementary Fig. S1).

### Statistical Analysis

R 4.1.1 and R Studio 1.4.1717 were used for statistical analysis. The data were first tested for normality of distribution. For 3 or more groups, Tukey’s HSD test was carried out after one-way analysis of variance (ANOVA) to analyze the significant differences (*P* < 0.05). The significant difference between two groups were analyzed by Student’s *t*-test (*P < 0.05, **P < 0.01). All data were shown as mean ± standard error (SE).

## Results

### Short-day Photoperiod Reduced JH Synthesis and JH-related Gene Expression in Diapause *C. nipponensis*

**Fig. 1.**
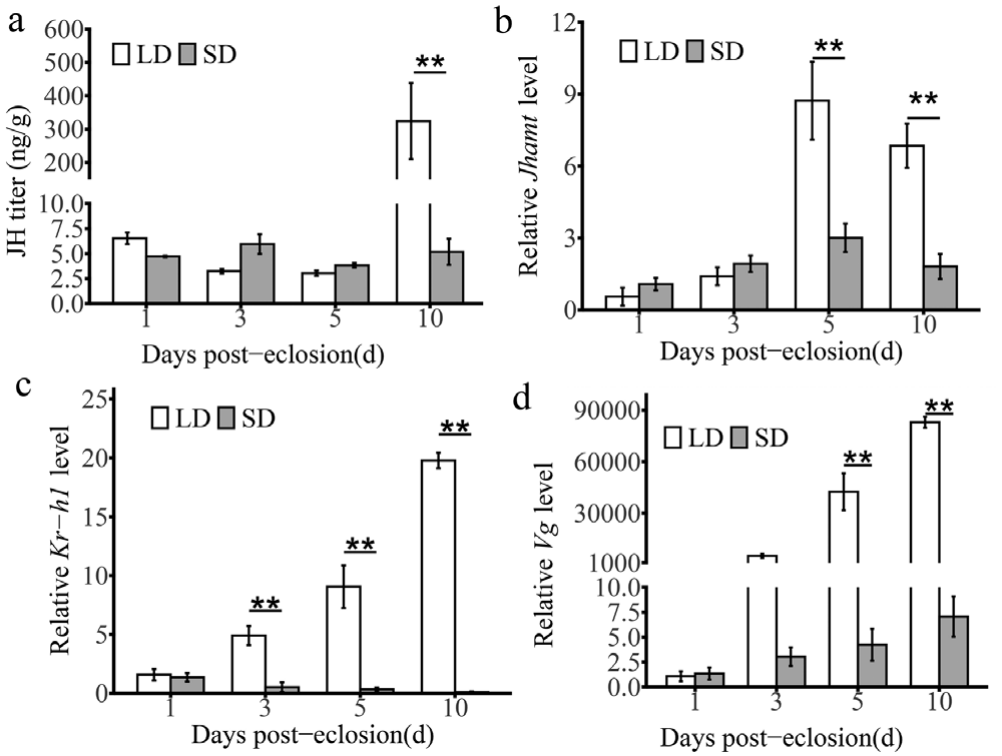
Effects of photoperiod on JH synthesis and JH-related gene expression in *C. nipponensis*. (A) The JH III (ng/g female lacewings) in *C. nipponensis* reared under LD and SD photoperiod. Expression profiles of *Jhamt* (B), *Kr-h1* (C), and *Vg* (D) in intact *C. nipponensis* reared under LD and SD photoperiod. Data are shown as mean ± SE. Student’s *t*-test was used to analyze the statistical significance of the difference between the means of the two treatment groups (*P < 0.05, **P < 0.01). *indicates that the gene expression of *C. nipponensis* is significantly different on the same day under the long and short photoperiods (*P < 0.05; **P < 0.01), LD: long-day (15:9 h light:dark); SD: short-day (9:15 h light: dark).

We determined JH titers and the expression of both JH synthesis genes and response genes in *C. nipponensis* reared under LD and SD photoperiod. The results showed that the JH titers exhibited no significant difference between LD and SD females at 1, 3, and 5 d of *C. nipponensis* after emergence (Fig. 1A). JH titers of LD females increased to 300-fold and significantly higher than that of SD females at 10 d of *C. nipponensis* after emergence (*F* = 7.84; df = 3,16; *P* < 0.001). A similar tendency (Fig. 1B to D) toward lower expression of *Jhamt*, *Kr-h1*, and *Vg* was seen in SD females. Compared with LD females (reproductive), SD females (diapause) showed that the expression of *Jhamt*, *Kr-h1*, and *Vg* was significantly lower at both 5 and 10 d of *C. nipponensis* after emergence (*Jhamt*: *F* = 12.81; df = 3,27; 5 d, *P* < 0.001; 10 d, *P* < 0.001. *Kr-h1*: *F* = 97.78; df = 3,29; 5 d, *P* < 0.001; 10 d, *P* < 0.001. *Vg*: *F* = 59.71, df = 3,29; 5 d, *P* < 0.001; 10 d: *P* < 0.001).

### SD Photoperiod Suppresses Females’ Ovarian Development and Promotes Triglyceride Accumulation in Diapause *C. nipponensis*

**Fig. 2.**
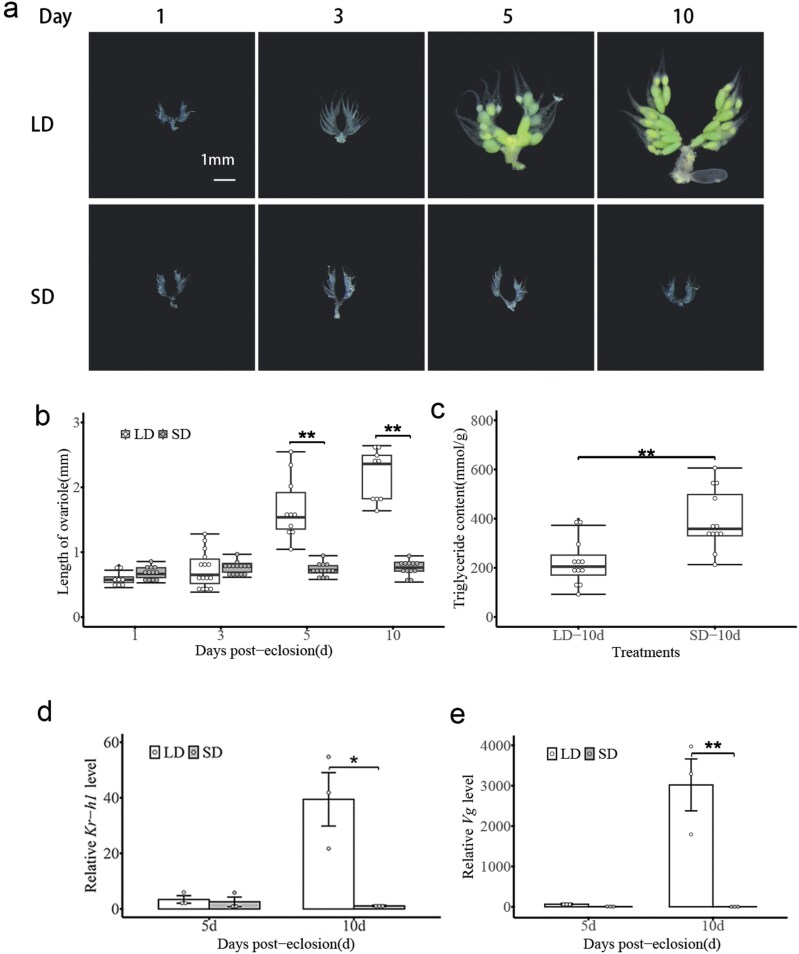
Effects of SD photoperiod on females’ ovarian development and lipid accumulation in *C. nipponensis*. The morphology of ovarian development (A) and the ovariole length (B) in *C. nipponensis* females at 1, 3, 5, and 10 d post-eclosion under LD and SD photoperiod. The triglyceride content in *C. nipponensis* females at 10 d post-eclosion under long and short photoperiods (C). Expression patterns of *Kr-h1* (D) and *Vg* (E) in the fat body of *C. nipponensis* females. Data are shown as mean ± SE. Student’s *t*-test was used to analyze the statistical significance of difference between the means of the 2 treatment groups. *indicates that the gene expression of *C. nipponensis* is significantly different in treatment groups (*P < 0.05; **P < 0.01), LD: long-day (15:9 h light:dark); SD: short-day (9:15 h light:dark).

We investigated the development status of ovarioles and ovaries of *C. nipponensis* females at 1, 3, 5, and 10 d after emergence under LD and SD photoperiod (Fig. 2A and B). The results showed that shortening the day length prevented ovarian development, resulting in significantly smaller ovaries (*F* = 50.97, df = 3,85; *P* < 0.001), but ovarian maturation of the LD females (reproductive) progressed normally. We then tested the effects of photoperiod on lipid accumulation of females at 10 d after emergence (Fig. 2C). The accumulation of triglyceride was significantly higher in diapausing lacewing compared with reproductive females (*t* = 3.86; df = 22; *P* < 0.001). Finally, we determined expression profiles of *Kr-h1* and *Vg* in the fat body of female adult lacewing under LD and SD photoperiod from 5 and 10 d after emergence (Fig. 2D and E). The results of qPCR revealed that *Kr-h1* expression levels in LD females were significantly increased by 40-fold at 10 d compared to the SD females (*F* = 14.18; df* *= 3,8; *P* = 0.001). Compared to LD adult females, the *Vg* transcription in the fat body was significantly lower in diapausing lacewing at 10 d after emergence (5 d: *F* = 21.66; df = 3,8; *P* = 0.99. 10 d: *F* = 21.66; df = 3,8; *P* < 0.001), showing developmental patterns similar to *Kr-h1*.

### JH III Induces Ovarian Development But Restrains Lipid Accumulation in Diapause Lacewing

**Fig. 3.**
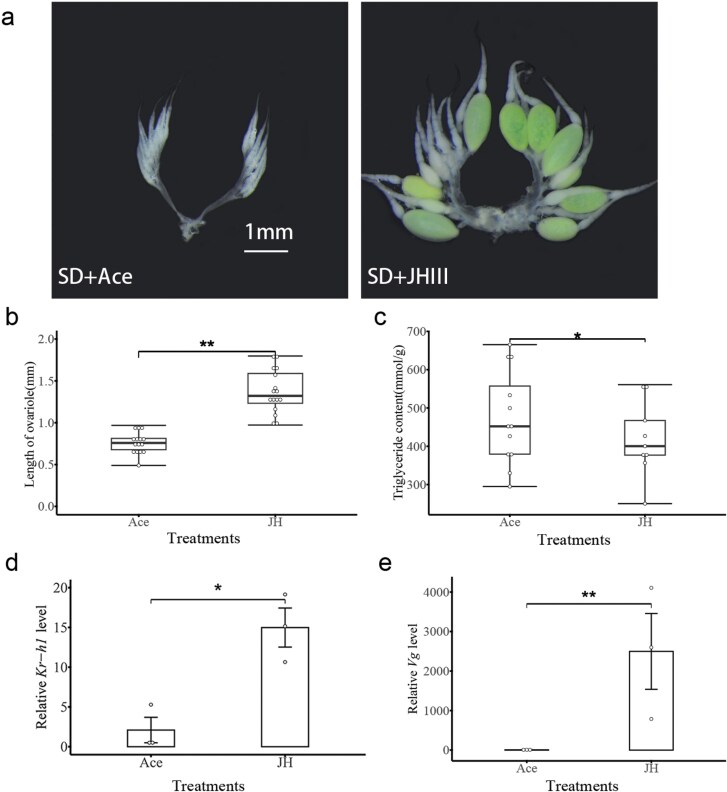
Effects of JHIII on female of *C. nipponensis*. Under the SD photoperiod, the morphology of the ovary (A), the ovariole length (B), and the triglyceride content (C) after exogenous JHIII treatment. Expression of *Kr-h1* (D) and *Vg* (E) in the fat body after exogenous JHIII treatment. Data are shown as mean ± SE. Student’s *t*-test was used to analyze the statistical significance of the difference between the means of the 2 treatment groups (*P < 0.05, **P < 0.01). LD: long-day (15:9 h light:dark); SD: short-day (9:15 h light:dark).

To test the function of JH in the regulation of reproductive diapause in *C. nipponensi*s*, the newly emerged SD females were exposed to exogenous JH* III. The treatment of synthetic JH III significantly increased the ovariole length (*t* = −8.20; df = 22; *P* < 0.001) (Fig. 3A and B), while the triglyceride content was significantly decreased (*t *= 2.14; df = 16; *P* = 0.048) (Fig. 3C). The exogenous JH III increased *Kr-h1* and *Vg* expression 15-fold and 2,000-fold in the fat body, respectively (*Kr-h1*: *t* = −4.40; df = 4; *P* = 0.011. *Vg*: *t* = −7.47; df = 2; *P* = 0.017) (Fig. 3D and E).

### The Knockdown of *Kr-h1* in Reproductive Females Exhibits a Diapause-like Phenotype

**Fig. 4.**
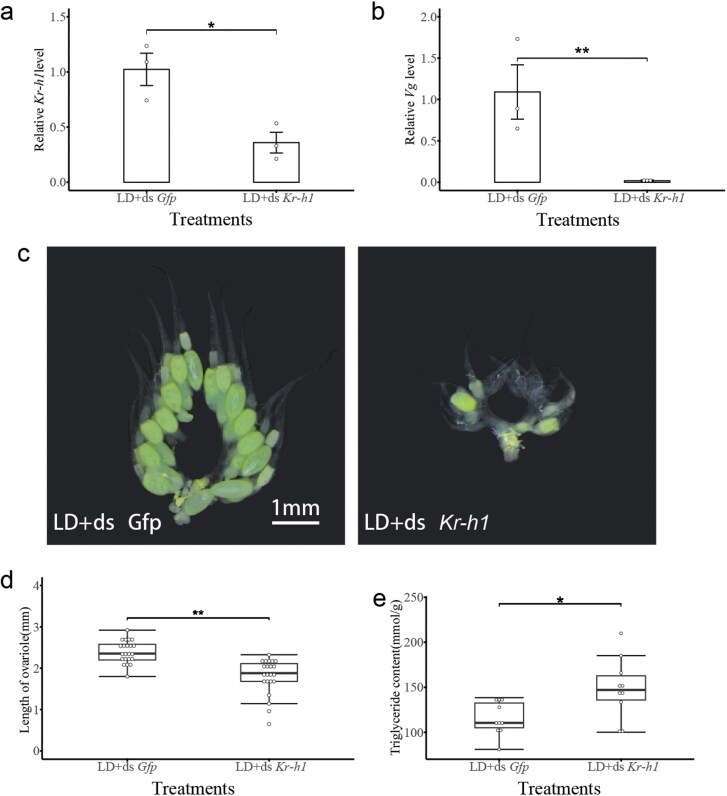
Effects of *Kr-h1* on diapause-related phenotype of *C. nipponensis*. Under the LD photoperiod, the effects of *Kr-h1*-interfering on the expression of *Kr-h1* (A) and *Vg* (B), the morphology of the ovary (C), the ovarian length (D), and the triglyceride content (E). Student’s *t*-test was used to analyze the statistical significance of the difference between the means of the 2 treatment groups (*P < 0.05, **P < 0.01). LD: long-day (15:9 h light:dark); SD: short-day (9:15 h light:dark).

**Fig. 5.**
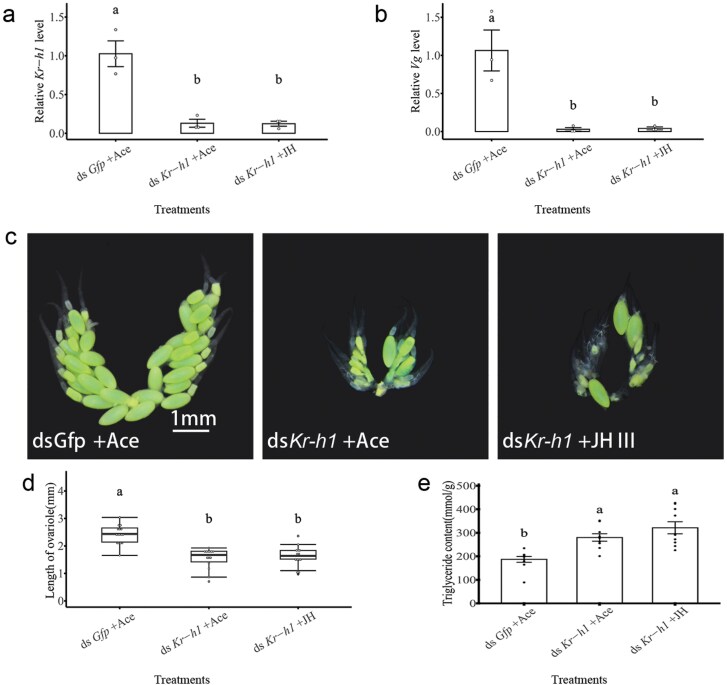
Effect of JH and *Kr-h1* on female of *C. nipponensis.* Under the LD photoperiod, after *ds-Kr-h1* injection, the effects of JHIII on the expression of *Kr-h1* (A) and *Vg* (B). The rescue of JHIII on the morphology of the ovary (C), the ovarian length (D), and the triglyceride content (E). Data are shown as mean ± SE. Student’s *t*-test was used to analyze the statistical significance of the difference between the means of the two treatment groups (*P < 0.05, **P < 0.01). LD: long-day (15:9 h light:dark); SD: short-day (9:15 h light:dark).

RNAi were used to investigate the function of *Kr-h1* in the reproductive process. Compared with the ds*Gfp* injection, the *Kr-h1* expression was significantly reduced by 54.21% after ds*Kr-h1* injection (*t* = 3.95; df = 5; *P* = 0.01) (Fig. 4A). The injection of ds*Kr-h1* notably downregulated *Vg* expression in the fat body (*t* = 3.7; df = 5; *P* = 0.014) (Fig. 4B). The interference of *Kr-h1* exhibits a diapause-like phenotype, including an increased triglyceride content and a blocked ovarian growth (ovariole length: *t* = 5.42; df = 42; *P* < 0.001. triglyceride content: *t* = −2.74; df = 18; *P* = 0.013) (Fig. 4C to E). Exogenous JH treatment could not enhance the expression of *Kr-h1* after *Kr-h1* interfering (*F* = 25.82; df = 2,6; *P* = 0.001) (Fig. 5A). Exogenous JH treatment on ds*Kr-h1*-injected females also could not stimulate *Vg* expression in fat body (*F* = 14.49; df = 2,6; *P* = 0.008) (Fig. 5B). The diapause-like phenotype, smaller ovarian and higher triglyceride content, was maintained after exogenous JH treatment (Ovariole length: *F* = 25.33, df = 2,40; *P* < 0.001. Triglyceride content: *F* = 14.02; df = 2,26; *P* < 0.001) (Fig. 5C to E).

## Discussion

In this work, we confirmed the JH represses reproductive diapause through its responding gene *Kr-h1* in the females of *C. nipponensis.* Our data revealed that long-photoperiod and exogenous JH induces *Kr-h1*, *Vg* expression, and ovarian growth, but suppresses triglyceride accumulation to inhibit diapause. Furthermore, interference of *Kr-h1* inhibited *Vg* expression, restrain ovarian development, but promoted triglyceride storage, thus matching the suppression of JH. Previous studies have shown that JH signaling performs diverse roles including in development and reproduction (Jindra et al. 2015, Li et al. 2019). Compared with reproductive females, we found that JH titers and the expression of JH-related genes were inhibited in diapause females. This corresponds to the study that lacking JH exhibit arrested ovarian development, upregulated the lipid storage and then enter reproductive diapause as in Colorado potato beetle (de Kort 1990, Denlinger 2002, Denlinger et al. 2012). Similar results have been demonstrated in *C. bowringi* and *Harmonia axyridis* (Tian et al. 2021, Gao et al. 2022a). On the other hand, applying JHs or JHA can terminate *C. pipiens* adult diapause (Kim et al. 2010, Denlinger et al. 2012). In this study, application of exogenous JH on diapause female adult lacewings induced ovarian growth and dropped triglyceride store. Likewise, topical treatment with JH analogs to diapause-destined females of *C. bowringi* and *H. axyridis* prompts ovarian development, and restrains lipid accumulation (Liu et al. 2016, Gao et al. 2022a, 2022b). The application of synthetic JH to *C. pipiens* diapausing females result in oocyte growth (Spielman 1974). Thus, those evidences indicate that JH is essential to terminate reproductive diapause.

*Kr-h1* has been shown to be associated with prevention of metamorphosis (Minakuchi et al. 2009, Konopova et al. 2011) and induction of reproduction (Smykal et al. 2014, Song et al. 2014). Our study suggested that *Kr-h1* may be a key component of JH pathway in repressing adult diapauses. First, the *Kr-h1* expression was upregulated by long photoperiod and was inhibited by SD condition. Nevertheless, there was no significant difference in JH titers between LD and SD individuals on 5 d post-eclosion, whereas there were significant differences in the expression of *Kr-h1* and *Vg*. This suggests that *Kr-h1* and *Vg* are regulated not only by JH but also by other factors, such as insulin/IGF signaling (Zhu et al. 2020). However, subsequent application of exogenous JH demonstrated that JH can indeed affect the expression of *Kr-h1* and *Vg*. Then, interfering *Kr-h1* expression reduced the expression of *Vg* genes in the fat body, arrested ovarian development, and also increased the triglyceride content of *C. nipponensis* females under long photoperiod. Similar functions have been studied in several insect species, such as *L. migratoria* (Song et al. 2014), *Tribolium castaneum* (Parthasarathy et al. 2010), *Bombyx mori* (Zhu et al. 2021), *P. americana* (Zhu et al. 2020), *Helicoverpa armigera* (Zhang et al. 2018), *Bactrocera dorsalis* (Yue et al. 2018), *Nilaparvata Lugens* (Jiang et al. 2017), *Sogatella furcifera* (Hu et al. 2020), and *H. axyridis* (Han et al. 2022). The previous studies have shown that exogenous JH terminates diapause in *Chrysoperla sinica* (Huang et al. 2021). We treated *Kr-h1*-depleted lacewing with exogenous JH III, but the treatment failed to rescue the reproductive development phenotypes. Thus, those evidences indicate that *Kr-h1* is one of the essential repressors of diapause termination regulated by JH adult diapause.

**Fig. 6.**
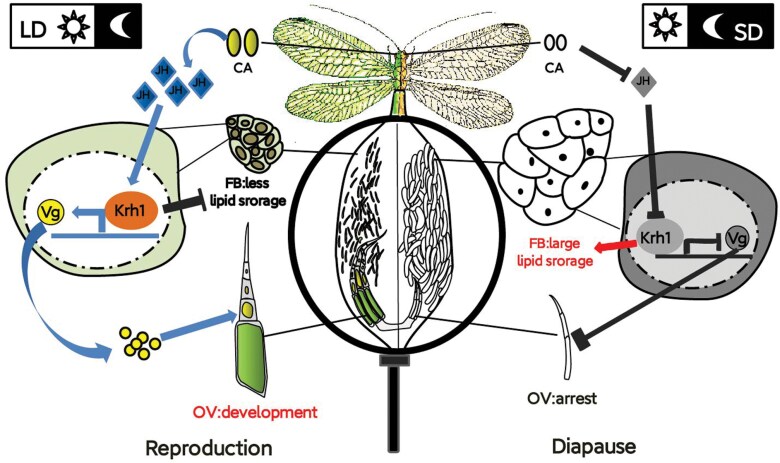
Schematic view of JH signaling and *Kr-h1* regulates reproduction and diapause in *C. nipponensis*. Under LD conditions, corpora allata (CA) synthesizes JH in *C. nipponensis* females, and then JH stimulates *Kr-h1* expression. Thus, *Kr-h1* promotes the ovary development but suppresses lipid accumulation in the fat body. Contrarily, under the SD, JH production is blocked, and *Kr-h1*-mediated JH signaling is then absent, thereby triggering diapause traits.

In the current research, further work on potential mechanism between JH-*Kr-h1* signaling and reproductive diapause in lacewing adults was lacking. In *C. bowringi*, the complex cross-talk between JH-Met-*Kr-h1* signaling and the lipolysis pathway is discussed. *Kr-h1* promotes triacylglycerol lipase expression (a gene downregulation of the lipolysis pathway) to suppress the diapause response (Guo et al. 2021). Our study demonstrated that, *Kr-h1* was identified as a key component of JH pathway in repressing adult diapauses. The schematic view of JH signaling in *C. nipponensis* during reproductive diapause is shown in Fig. 6. Under the LD conditions, corpora allata synthesizes JH in *C. nipponensis* females, then JH stimulates *Kr-h1* expression. Thus, *Kr-h1* promotes the ovary development but suppresses lipid accumulation in fat body. Contrarily, under the SD, JH production remains inactive, and *Kr-h1*-mediated JH signaling is then absent, thereby triggering diapause traits. This work shows the critical role of *Kr-h1* in regulating JH signaling to promote reproduction. However, how JH signaling regulates the nutrient metabolic switching to lipid storage in reproductive diapause of *C. nipponensis* should be examined in future studies.

## Supplementary Material

ieaf027_suppl_Supplementary_Figures_S1
