# Supplementary figures and images for: Krüppel homolog 1 mediates juvenile hormone action to suppress photoperiodic reproductive diapause-related phenotypes in the female Chrysoperla nipponensis (Neuroptera: Chrysopidae)
Source: J Insect Sci. 2025 Mar 21;25(2):7. doi: 10.1093/jisesa/ieaf027 (PMC11926538; doi:10.1093/jisesa/ieaf027)

**
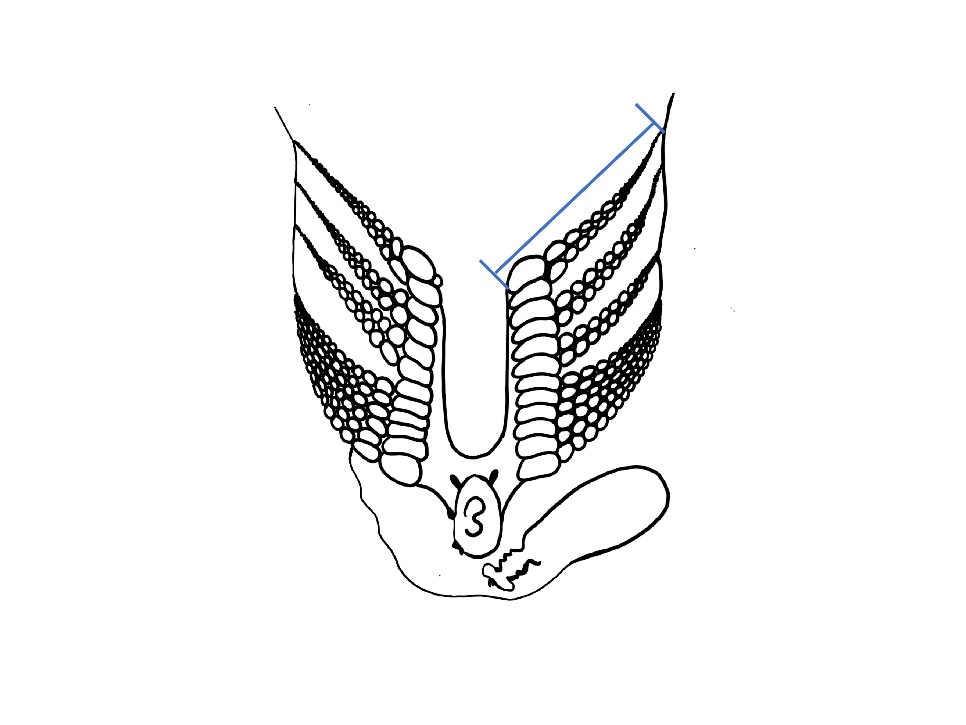
**

**Fig. S1.** Measure of ovarioles length of the ovary

Supplement: ieaf027_suppl_Supplementary_Figures_S1 [file ieaf027_suppl_supplementary_figures_s1.docx]
